# Effectiveness of repetitive transcranial magnetic stimulation (rTMS) for the treatment of obsessive-compulsive disorder (OCD): a meta-analysis

**DOI:** 10.1192/bjo.2021.118

**Published:** 2021-06-18

**Authors:** Kabir Garg, Naomi Fineberg, Luca Pellegrini, Arun Enara, Eduardo Cinosi

**Affiliations:** 1 Oxleas NHS Foundation Trust; 2 Hertfordshire Partnership University NHS Foundation Trust, University of Hertfordshire; 3 Hertfordshire Partnership University NHS Foundation Trust; 4 Hertfordshire Partnership University NHS Foundation Trust

## Abstract

**Aims:**

OCD is a chronic and debilitating psychiatric illness. Current first-line treatments include serotonin reuptake inhibitors and cognitive behavioural therapy, but a substantial minority of patients fail to respond adequately, requiring further forms of intervention usually provided in a sequenced algorithm. Repetitive Transcranial Magnetic Stimulation (rTMS) uses magnetic pulses passed through a coil placed on the scalp to stimulate the underlying brain region. Clinical trials of r-TMS in OCD have produced conflicting results, possibly related to the variability in the site of stimulation, protocols used, and variability in the selection of patients. We perform an updated systematic review and meta-analysis of the effectiveness of rTMS for the treatment of OCD aimed to determine whether certain rTMS parameters (i.e. site, duration, protocol etc.) or patients’ characteristics (i.e age, duration of illness, illness severity, treatment resistance etc), are associated with effectiveness. Our overarching aim is to determine the place of rTMS in the sequenced OCD care-pathway.

**Method:**

The meta-analysis is pre-registered in PROSPERO (ID: 241381). Potentially relevant studies will be retrieved using the MEDLINE, PsycINFO, and Cochrane Library databases using the parameters [‘obsessive compulsive disorder’ or ‘OCD’ or ‘obsessions’ or ‘compulsions’] AND [‘transcranial magnetic stimulation’ or ‘TMS’]. The reference lists of retained articles will also be scrutinized for additional relevant publications. Only full text English language articles will be included in the review. The methodological quality of the studies will be assessed using CONSORT criteria. A summary of the study's quality as a randomized clinical trial will be produced.

**Result:**

Our preliminary analysis shows some efficacy for r-TMS in non-treatment resistant OCD than treatment resistant OCD. Detailed results will be presented in the poster at the event. Effect measure will be either categorical (e.g. relative risk (RR) or odds ratio (OR) or continuous (mean difference or standardized mean difference - Hedge's g or Cohen's d - when taking into consideration the severity of the disorder as a dimension). These outcomes will be measured through validated instruments, in the form of both self- rated scales and observer rated scales including semi-structured interviews.

**Conclusion:**

This meta-analysis will identify the patient, illness and protocol parameters that determine clinical outcomes, as guide to optimizing the role of rTMS in the care of patients with OCD.

